# Decentralized environmental applications of a smartphone-based method for chemical oxygen demand and color analysis

**DOI:** 10.1038/s41598-023-37126-9

**Published:** 2023-07-08

**Authors:** Jussara Câmara Cardozo, Inalmar D. Barbosa Segundo, Edney R. V. P. Galvão, Djalma R. da Silva, Elisama V. dos Santos, Carlos A. Martínez-Huitle

**Affiliations:** 1 Renewable Energies and Environmental Sustainability Research Group, Institute of Chemistry, Federal University of Rio Grande do Norte, Av. Salgado Filho 3000, Lagoa Nova, Natal, RN CEP 59078-970 Brazil; 2 Departament of Petroleum Engineering, Federal University of Rio Grande do Norte, Av. Salgado Filho 3000, Lagoa Nova, Natal, RN CEP 59078-970 Brazil; 3 School of Science and Technology, Federal University of Rio Grande do Norte, Av. Salgado Filho 3000, Lagoa Nova, Natal, RN CEP 59078-970 Brazil; 4 National Institute for Alternative Technologies of Detection, Toxicological Evaluation and Removal of Micropollutants and Radioactives (INCT-DATREM), Institute of Chemistry, UNESP, Araraquara, SP CEP 14800-900 Brazil

**Keywords:** Pollution remediation, Environmental monitoring, Electrocatalysis

## Abstract

This study is focused on a proposal of a smartphone imaging-based quantification for providing a simple and rapid method for the analysis of chemical oxygen demand (COD) and color throughout the use of the HSV and/or RGB model in digital devices. For COD, calibration curves were done based on the theoretical values of potassium biphthalate for a proper comparison between the spectrophotometer and the smartphone techniques. The smartphone camera and application attain an average accuracy higher than the analysis in the spectrophotometer (98.3 and 96.2%, respectively). In the color analysis, it was demonstrated that only the UV–vis bands measurement is not feasible to perform the real abatement of the dye in the water because the limiting concentration that allows obtaining a linear relationship in this equipment related to the dye concentration is about 10 mg L^−1^. Above this value, the spectrophotometer can not reach the real difference of color in the solution. Meanwhile, the smartphone method by using the camera reaches linearity until 50 mg L^−1^. From an environmental point of view, smartphones have been used for monitoring several organic and inorganic pollutants, however, no attempts have been published related to their use to evaluate the color and COD during wastewater treatment. Therefore, this investigation also aims to assess the utilization of these methods, for the first time, when high-colored water polluted by methylene blue (MB) was electrochemically treated by using a boron-dopped diamond (BDD) as the anode, with different current densities (*j* = 30, 45, 60, and 90 mA cm^−2^). COD and color abatement results clearly showed that different organic matter/color removal efficiencies were achieved, depending on the *j* used. All the results are aligned with the studies already available in the literature, with the total removal of color in 120 min of electrolysis with 60 and 90 mA cm^−2^, and almost 80% of COD abatement with the higher *j*. Moreover, samples of real effluent from beauty salons were compared, with standard deviation varying from only 3 to 40 mg O_2_ L^−1^, which is acceptable for COD values close to 2000. Finally, the methods here presented can be a great benefit for public water monitoring policies, since it is cheap and has a decentralized characteristic, given that smartphones are very common and portable devices.

## Introduction

Stringent environmental regulations have been developed due to the constant increase of industrial activity, and consequently higher negative effects on the ecosystems. Pollutant emission control in waterbodies is an essential means of public governance, however, the impact of stricter environmental laws on the quality of social development is ambiguous, once the necessary analytical methods generally demand the use of specific and expensive equipment. Then, the government centralizes the monitoring procedures in specific centers to diminish the costs, of acquisition and maintenance. All these factors make the analytical processes more dilatory, mainly in undeveloped and developing countries.

A recent study has demonstrated that more rigorous environmental protocols induce technological innovations^1^. In this perspective, the use of smartphones-based technologies has increased in the most diverse areas, from health^2^ to environmental sciences^3^; and it can embrace billions of users around the world because these devices are portable, and generally has easy-to-use tools, such as convenient touch-screen display, high-resolution camera, useful processors, and large data storage capacity^4^. Among the methods already proposed in the literature, the simplest are colorimetric, where the smartphones’ cameras coupled with specific applications are responsible for detecting the color change and can substitute the use of expensive spectrophotometers, simplifying the analytical procedure without compromising the results^5,6^.

In the case of wastewater effluents, these can be characterized by pollution indicators such as biochemical oxygen demand (BOD), chemical oxygen demand (COD), suspended solids, color, toxicity, and turbidity^7,8^, which are parameters used worldwide as part of many directives dealing with water quality^9,10^. Regarding the industrial textile wastewaters, their composition can be highly complex where various chemical compounds can be present, such as dyes, surfactants, heavy metals, additives, pH adjustment chemicals, etc^11,12^. Then, the main aspects related to their treatment are considered the removal of colors and the organic load in order to accomplish the levels of environmental legislation. In the case of COD, it is a key parameter to assess surface water and wastewater quality. Generally speaking, it is defined as the number of oxygen equivalents consumed in the oxidation of organic compounds, required to oxidize matter using a chemical oxidizing agent in the conventional method, normally the dichromate ion (Cr_2_O_7_^2−^)^13^. During the process of sample digestion, chromium(VI) is reduced to chromium(III), and so it is possible to observe changes in the color of the solution since both species are colored and absorbed in the visible region of the spectrum.

In order to give timely information about the breadth and amount of pollution, there is an increasing demand for low-cost devices that can detect and monitor environmental contaminant concentrations quickly, easily, and on site (decentralized treatments or water quality analysis)^14^. The ultimate goal is to make it possible for each person to carry a personal laboratory with them at all times. In the last years, the use of smartphones for analytical reasons has exponentially increased. This little and practical device has transformed many aspects of our lifes and made it possible to use it as a point-of-care diagnostic tool as well as a biosensing and environmental monitoring device. It also offers easy wireless interface with other devices^14^ and specially in the health and environmental applications. In the latter, the demand for portable imaging systems has increased due to activities like pathogen screening and water quality monitoring in order to guarantee water quality and reuse, considering that wastewater treatment facilities utilize the proper procedures. In this perspective, the creation of tiny, decentralized systems seems like a potential solution for some sectors as an alternative sustainable water resource to fillin the Sustainable Development Goal 6. Whitin this frame, no attempts utilizing a smartphone to assess the water decontamination have been reported in the literature. Then, the objective of this work is to create a decentralized analytical approach based on the usage of a smartphone imaging-based quantification for providing a simple and rapid method for the analysis of chemical oxygen demand (COD) and color throughout the use of the HSV and/or RGB model in digital devices. The findings from this investigation may be used to create and modify a portable measurement tool that can be used directly in a “decentralized” futuristic supply model, where local water reuse or water treatment technologies are added to already existing municipal water delivery systems.

To do that, the discoloration and degradation levels during the electrochemical treatment, as an advanced oxidation process, of a synthetic waste solution containing a basic water-soluble dye (methylene blue) was evaluated by using a smartphone as an analytical tool. The results of the analyses are obtained by the colorimetric changes of the samples captured by the camera of the smartphone, which can be directly measured by a color analysis application. The characteristic color can be represented by the RGB model, where the values of R (red), G (green), and B (blue) describe the scanned color of the selected area^15,16^, or even by the HSV model (hue, saturation, and value). The data obtained were also compared with analytic instrumentation to validate the protocol proposed here.

## Experimental

### Measurements of COD and color using spectrometry and image acquisition

**Figure 1:**
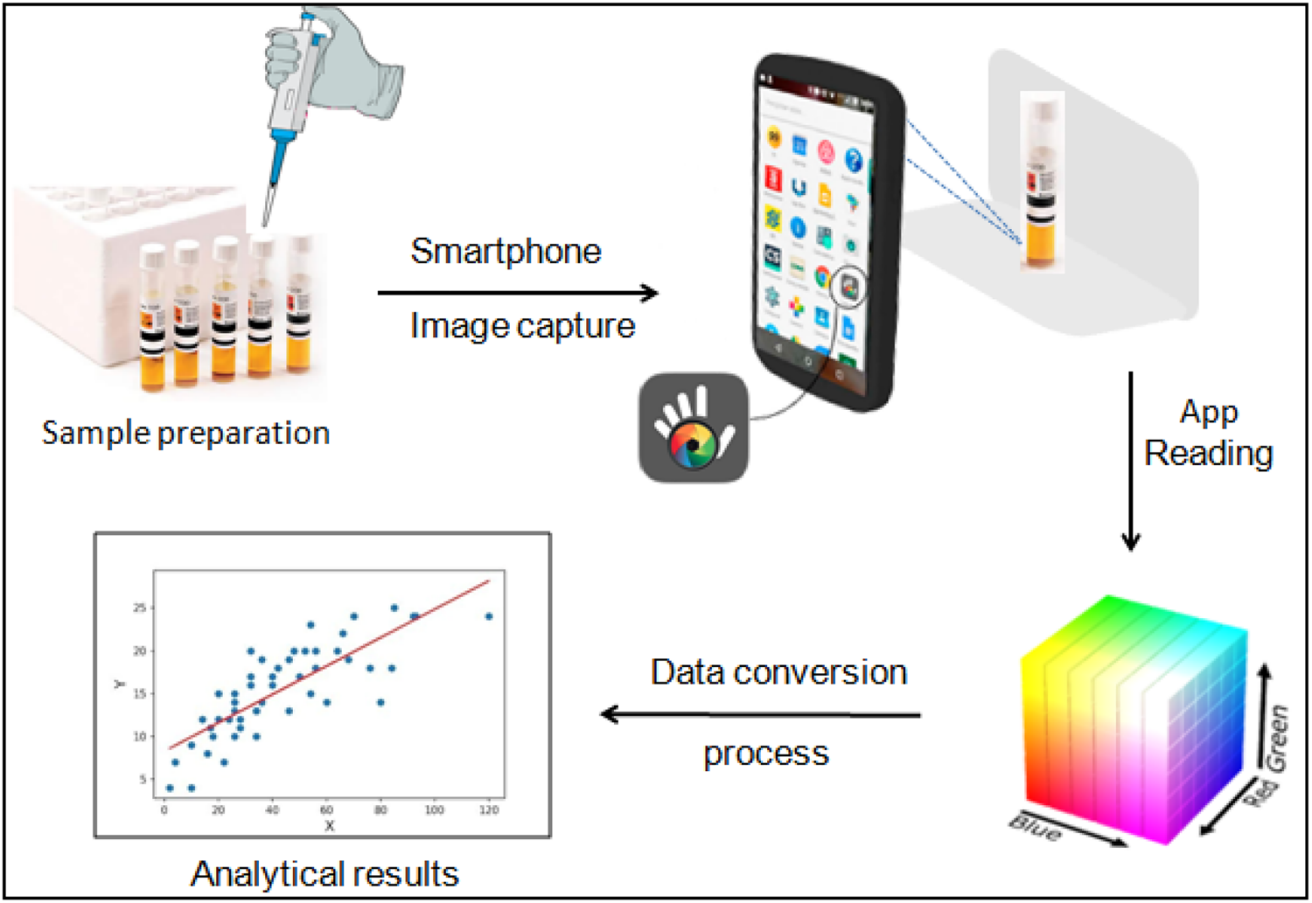
Illustration of the proposed analytical process.

The linearity of the developed method was verified through the analysis of the calibration curve using the conventional method to obtain the COD values. For the calibration curve, potassium biphthalate was used. The tubes containing each point of the analytical curve were digested in a thermal reactor (HANNA, HI839800), at a temperature of 150 °C for 2 h. Low-range HANNA kits were used considering the concentration of the samples (0–150 mg O_2_ L^−1^). Since it is a colorimetric procedure, the interferents of the presented methodology are the same as the HANNA kits. After cooling the samples, the different procedures of quantification of the analyte were performed, using a spectrophotometer (model Specord 210 Plus, Analytik Jena) and by the capture of images by a smartphone (Motorola Moto G^5S^ Plus with 13 megapixels), equipped with Android 8.1 and using the free accessibility application Color Grab (version 3.7.7, 2020 Loomatix©) without the use of flash. The area of image capture, illumination, and focal distance was kept constant in all experiments (the experimental setup is briefly shown in Fig. 1). For this, it was used the same artificial cold light for illumination (with the lamp located just above the place, to avoid shadows), a clean white background 5 cm behind the sample, and the smartphone camera ready to capture at a linear height pointed to the middle of the sample, separated 10 cm from it. After the capture, the values of the individual components (RGB and HSV) give the mean values for all pixels. For the HSV method, only the percentage of saturation is sufficient for the analysis, which represents the color intensity and its purity degree through the radial coordinate. For RGB, all data were transformed into greyscale intensities, based on the information reported in the literature, according to Eq. (1)^15^.

1 $${\text{I}} = 0.299{\text{R}} + 0.587{\text{G}} + 0.114{\text{B}}$$

where I is the brightness intensity in greyscale, and R, G and B are variables that characterize the color of the work area (using the RGB model).

For comparison purposes between the methods, the intensity of light given as a response was converted into absorbance using the Lambert–beer Eq. (2) to minimize the errors that result from obtaining images with slight variations in brightness caused by the absorption of light by the chromophore molecules present in the color solution.

2 $${\text{A}} = - \log \frac{{\text{I}}}{{{\text{I}}_{0} }}$$

where (I) is the final value of the greyscale intensity and (I_0_) is the blank solution captured by the smartphone camera. Several experiments and analysis were replicated to minimize the experimental error, with a confidence level of > 95% for all determinations, but only one set of data was chosen as the best.

### Reagents

Methylene blue (C_16_H_18_N_3_SCl, 319.85 g mol^−1^, purchased by Reagen) was used for the calibration curve and all the oxidation assays. Sodium sulphate used as background electrolytes was supplied by Synth. The potassium biphthalate (C_8_H_5_KO_4_, 204.22 g mol^−1^), used for the COD calibration curve, was acquired by Êxodo Científica. All solutions required for analytical determinations were prepared with distilled water.

### Electrochemical systems

The electrochemical oxidation system was performed in galvanostatic conditions using a batch cell with 400 mL of solutions containing 50 mg L^−1^ MB in 0.1 M Na_2_SO_4_. The cell contained boron-doped diamond (BDD) as the anode and titanium as the cathode. The total surface area of the anode is 4 cm^2^. The electrolysis was carried out using a power supply by applying different current densities (30, 45, 60, and 90 mA cm^−2^) for 2 h. During the electrochemical experiment, samples were collected and subjected to a COD and color removal analysis using image capture by smartphone, as described in 2.1. Also, spectrophotometric measurements were obtained for comparison. Several experiments and analysis were replicated to minimize the experimental error, with a confidence level of > 95% for all determinations, and the error bars were included to visualize the variability of the plotted data.

### Analytical procedures

The decolorization of methylene blue dye solutions was followed by image acquisition and their color changes were determined by a color analysis application with a smartphone.

In the case of color removal, the percentage of decolorization efficiency was then estimated by Eq. (3):

3 $${\text{Color}}\;{\text{removal}} \left( \% \right) = \frac{{{\text{I}}_{0} - {\text{I}}_{{\text{t}}} }}{{I_{0} }} \times 100$$

where I_0_ and I_t_ are the color intensity at the initial time and time *t* of electrolysis.

Meanwhile, the oxidation of dye solutions was monitored from their COD values, estimating the percentage of COD removal by Eq. (4):

4 $${\text{COD}}\;{\text{removal }}\left( \% \right) = \frac{{{\text{COD}}_{0} - {\text{COD}}_{{\text{t}}} }}{{{\text{COD}}_{0} }} \times 100$$

where COD_0_ and COD_t_ are the COD values at the initial time and time t of electrolysis.

Total current efficiency (TCE in %, Eq. 5) and energy consumption (EC, kWh COD m^−3^, Eq. 2) of the electrochemical treatment were also estimated according to the following equations:

5 $${\text{TCE }}\left( \% \right) = {\text{FV}}\left( {\frac{{({\text{COD}}_{0} - {\text{COD}}_{{\text{f}}} }}{{8{\text{I}}\Delta {\text{t}}}}} \right) \times 100$$

where COD_0_ and COD_f_ are initial and final chemical oxygen demands in g O_2_ L^−1^, respectively; F is the Faraday constant (96,487 C mol^−1^), V is the electrolyte volume (L), *I* the current (A), 8 is the oxygen equivalent mass (g eq.^−1^) and Δt is the total time of electrolysis, allowing for a global determination of the overall efficiency of the process.

6 $${\text{Energy}}\;{\text{consumption}} = \left( {\frac{{(\Delta {\text{E}}_{{\text{r}}} \times {\text{I}} \times {\text{t}}}}{{3600 \times {\text{V}}}}} \right)$$

where ΔEc (V) and *I* (A) are the average cell voltage and the electrolysis current, respectively; t is the time of electrolysis (s); and V is the sample volume (mL).

## Results and discussion

### COD analysis

Standard samples of potassium biphthalate (KHP) were analyzed according to three different analytical protocols: (1) the usual method^17^, with the use of a spectrophotometer; and with the smartphone’s camera, through (2) the HSV model; or (3) the RGB intensities.

**Figure 2:**
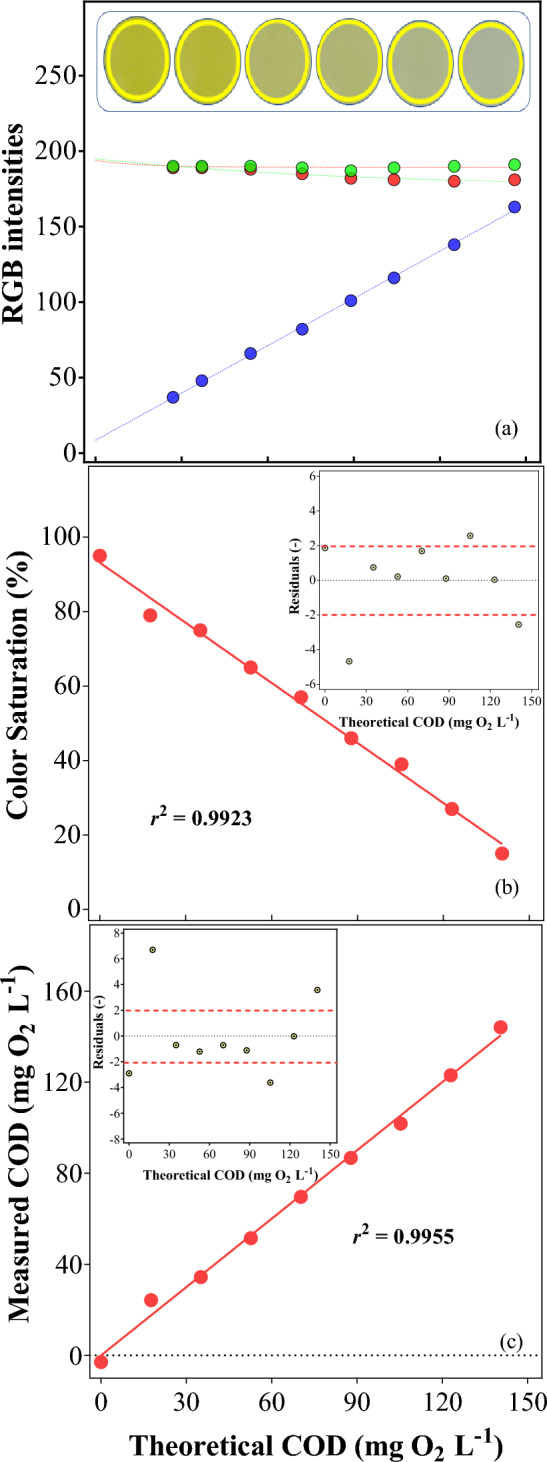
RGB color intensities for KHP standards, with the real images of the samples (**a**), calibration curves of the saturation captured by the smartphone (**b**), and spectrophotometer values (**c**). Insets: residuals of the respectives’ regressions.

Calibration curves were then performed by plotting the theoretical values of the COD of KHP, with the values obtained by the spectrophotometer and also the figures obtained by the smartphone at both HSV and RGB methods. Unfortunately, the RGB model was not suitable for the COD’s calibration curve, once the yellowish color of the digested samples makes unfeasible the correct procedure for transformation from the RGB values to the greyscale since differences are perceptible only in the blue intensity (Fig. 2a). Despite of it, the value of the saturation by the HSV model showed a good correlation with the digested potassium biphthalate standards (Fig. 2b, r^2^ > 0.99). Despite the good *r*^2^ values in both curves and the results from residuals demonstrating acceptable linearity around 0 (Fig. 2, insets of b and c), some residual points in both curves are a little far. It probably occurs due to small errors during the preparation of the standards, or even intrinsic errors from the used equipment. However, these outlined points did not contribute to a significant deviation, which can be noticed by the good linear regression. It also is good to point out that both curves were prepared in duplicate, with very similar results.

Table 1 presents the data of all calibration curves correlated with the theoretical COD of KHP standards. The COD results from the spectrophotometer show a considerable error, mainly in the lower ranges (0 and 15 mg KHP L^−1^). Thus, a calibration curve based on the theoretical values and the results of the spectrophotometer was performed for a more suitable comparison between the methods, once the same occurred with the use of the smartphone. Analyzing the results obtained, the most significant deviation was achieved at only one standard concentration, which was 15 mg KHP L^−1^, and it should represent an actual value of about COD of 17.6 mg O_2_ L^−1^. Nevertheless, it reached a value of 27 and 26.3 mg O_2_ L^−1^ by the spectrophotometer and the smartphone, respectively. Despite the deviation described above, the average accuracy of the methods reached good-quality results, attaining 96.2% for the measurements with the spectrophotometer, and 98.3% for the smartphone-based. Then, it is possible to observe that the COD determinations from the HSV color analysis smartphone application were extremely efficient compared to the measurements achieved by the conventional spectrophotometric method.

**Table 1:** Data of COD calibration curves.

C_8_H_5_KO_4_ (mg L^−1^)	Theoretical COD	COD^a^	COD^b^	Color saturation (%)	COD^c^	Average COD^d^	Standard deviation^d^
0	0.0	0	− 2.9	95	− 3.5	− 2.1	1.86
15	17.6	27	24.3	79	26.3	22.7	4.56
30	35.1	37	34.4	75	33.7	34.4	0.71
45	52.7	54	51.5	65	52.3	52.2	0.62
60	70.3	72	69.6	57	67.2	69.0	1.63
75	87.8	89	86.7	46	87.6	87.4	0.60
90	105.4	104	101.8	39	100.6	102.6	2.49
105	123.0	125	123.0	27	122.9	123.0	0.02
120	140.5	146	144.1	15	145.2	143.3	2.45

^a^Value at the Spectrophotometer (mg O_2_ L^−1^).

^b^Value corrected by calibration curve (theoretical COD × COD at the spectrophotometer, in mg O_2_ L^−1^).

^c^Value after calibration curve of Saturation (HSV model).

^d^Calculated from theoretical COD, COD^b^, and COD^c^*.*

### Color analysis

**Figure 3:**
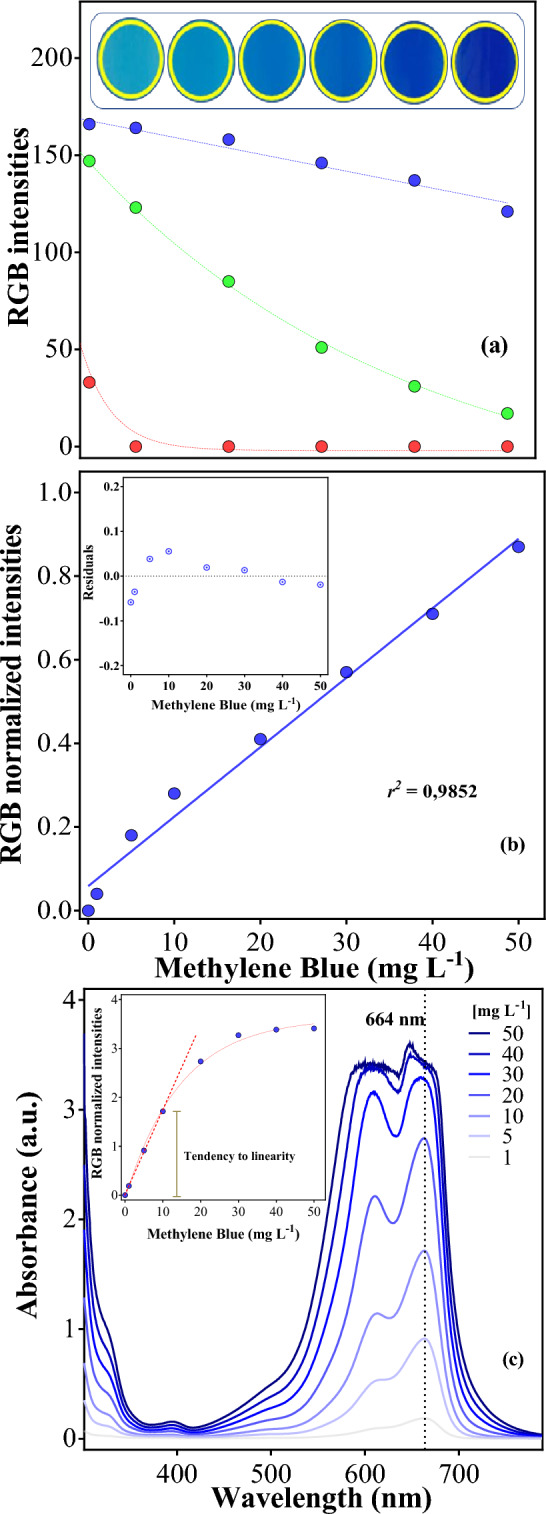
RGB color intensities for methylene blue dye standards (**a**); calibration curve of the RGB values captured by the smartphone (**b**, inset: residuals of the respectives’ regressions); and the absorbance spectrum of the standards (**c**, inset: intensity of the samples at 664 nm).

Aiming to prove the effectiveness of the smartphone-based color measurement even in high colorful samples, methylene blue (MB) was used as the dye for the measurements in this work. From a highly saturated solution (50 mg L^−1^), calibration curves were performed in the same way as the calibration for COD (Fig. 3).

Unlike the COD measurement, the analysis of color by the RGB values was achievable because these three values exhibited differences among the standards (Fig. 3a). Each RGB value was converted to a brightness intensity in greyscale according to Eq. (1), and normalized by Eq. (2). Then, both the RGB and the spectrophotometer values were plotted (Fig. 3a) and the curves are shown in Fig. 3b and c (inset).

The spectrophotometric analysis at 664 nm showed that the absorbance preseted a linear relationship with the concentration until 10 mg L^−1^ (Fig. 3c). In fact, if the concentration of the solution increases significantly, more than 10 mg L^−1^; the linearity is not obeyed (see inset in the Fig. 3c). This behavior is due to that the molecules are too close and consequently, the irradiation may not pass effectively through the solution. Conversely, a linear tendency is definitely observed with the smartphone-based method (Fig. 3b) in a concentration range from 0 to 50 mg L^−1^ because the changes on the color of the standard solutions are efficiently determined only with the analysis of their surface’s color. Instead, by using the spectrophometer, there is an specific instrumental absorbance’s limit that is not recommended to be ultrapassed. Thus, the smartphone protocol can be an effective alternative to perform the analysis of color abatement in water contaminated with MB dye.

**Figure 4:**
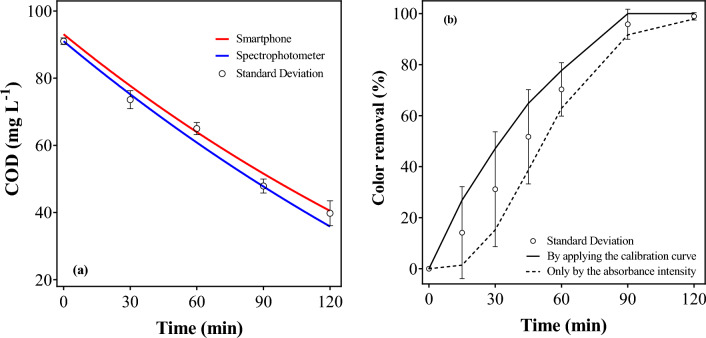
(**a**) Comparison of COD abatement with the smartphone-based and the spectrophotometer methods during 120 min of electrolysis with a *j* = 60 mA cm^−2^; and (**b**) the difference of the tendency of color removal (%) by applying the calibration curve, and by using only the absorbance intensity.

Several works in existing literature use the MB as a model compound for studying the degradation rates of specific treatment methods. In these works, the initial concentration of the pollutant usually varies from 10^18^ to 50 mg L^−1^^19^. According to the calibration curve here displayed (Fig. 3), the linearity of the spectrophotometer is only possible until 10 mg L^−1^. In fact, some authors have critically demonstrated that a dilution is needed because the most concentrated samples do not obey the Lambert–Beer lay in the range of 10–50 mg MB L^−1^^20^. Nonetheless, the great majority of the authors only calculated the removal in terms of percentage, based on the initial absorbance values and the value at a determined time *t*^19^. In this case, huge errors can occur in estimating the concentration, once from 20 to 50 mg MB L^−1^ no significant difference is demonstrated in the spectrophotometric values. In other words, by using only this raw data to calculate the removal of color, the researchers can find a huge initial error, where the color abatement is much lower than the real removal (Fig. 4b). For this reason, the smartphone-based method for analysing color, proposed in this work, can be a good choice for *in-loco* measurements of color abatment. Then, as a-proof-of-concept, EO was selected as AOP to depollute effluents, monitoring COD and color decay using smartphone protocol proposed here.

### Environmental application of the proposed methods through electrochemical advanced oxidation treatment

Advanced oxidative processes (AOPs) and electrochemical AOPs (EAOPs) are widely used as technologies for removing synthetic dyes from water. This theme started to be in evidence in the ’90 s but was in the ’00 s that a huge number of researchers starts to explore it in a further way^21^, mainly focusing on the properties of different anodic electrodes. In this work, a preliminary test was employed aiming to verify the efficacy of the smartphone-based method to follow de decaying of COD and dye concentration in a high-saturated MB solution (Fig. 4).

**Figure 5:**
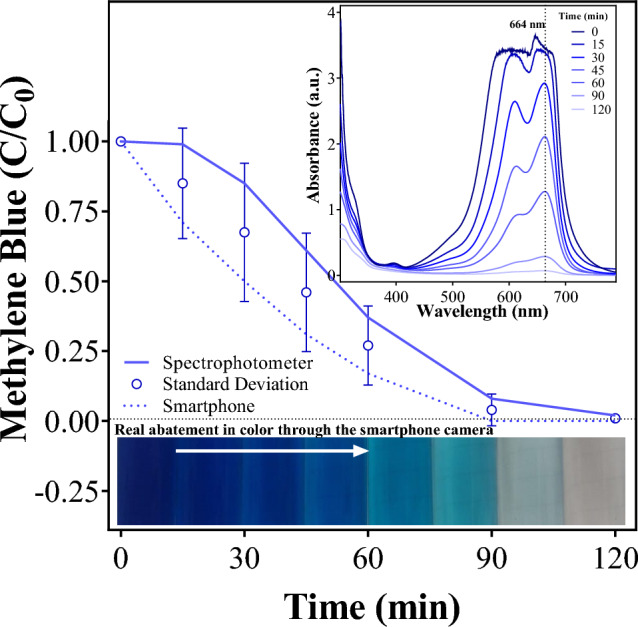
Timely decrease of absorbance at 664 nm and absorbance spectrum (inset) during MB electrolysis.

The standard deviation between the methods of COD analysis is constant even in the lowest concentration, corresponding to only 3.7 mg O_2_ L^−1^ at the time of 120 min. This behavior is predicted by the calibration curves (Table 1), which is adequate to guarantee that the smartphone-based method has a similar performance to the spectrophotometer equipment. Meanwhile, a comparison between the data obtained using the color calibration curve and the absorbance intensity demonstrated to be different. This behavior is clearly observed when smartphone and spectrophotometric measurements are compared via the colored-images obtained, as a function of electrolysis time (Fig. 5), by applying *j* = 60 mA cm^−2^ to treat a solution 50 mg MB L^−1^. As can be observed, the instrument absorbance limit imposes restrictions to achieve reliable data at higher MB concentration (see inset in the Fig. 5) achieving a similar color decay values within of 20 min of electrolysis (see full circles curve in the Fig. 5), as demonstrated above in Fig. 4b and in the previous discussion. However, the real color abatement is obtained by using the smartphone camera and the RGB analysis, demonstrating that the results are more valid figures (see Fig. 5).

**Figure 6:**
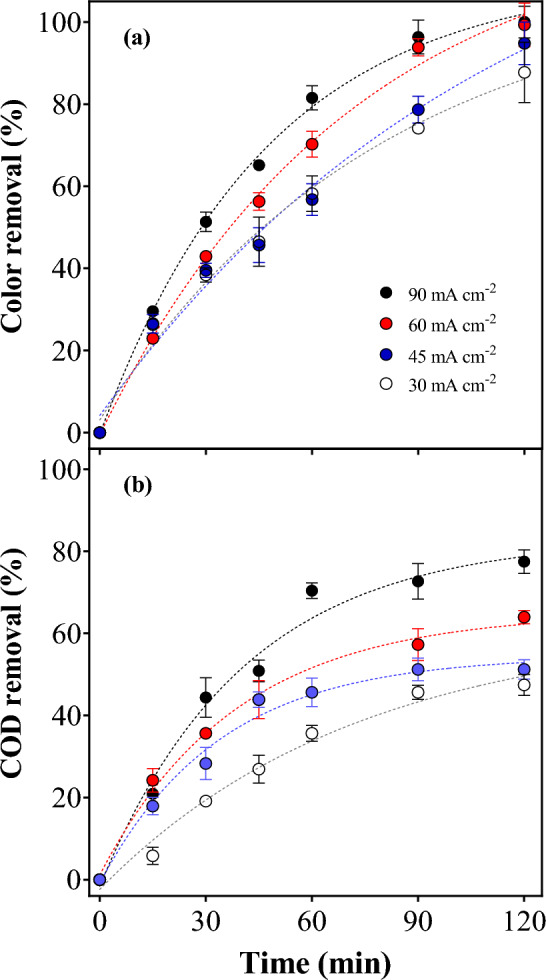
Color (**a**) and COD (**b**) abatement during 120 min of electrolysis by applying 30, 45, 60, and 90 mA cm^−2^.

In order to complete the applicability of the smartphone protocol, different current densities were tested aiming to find the better operating conditions to promote the elimination of organic matter, in terms of COD, and the decolorisation of a solution containing 50 mg MB L^−1^ (Fig. 6).

At 120 min of treatment, both the *j* of 60 and 90 mA cm^−2^ attained similar color removals, corresponding to 99 and 100%, respectively (Fig. 6). Conversely, 95 and 89% of color elimination were achieved at 45 and 30 mA cm^−2^, respectively. This behavior is due to the fragmentation of the cromophore group at MB molecule, which gives the color features of the solution. As previously showed in Fig. 5, the colored-images demonstrated that the descolorisation is attained, as a function of time, as a consequence of the attack of the oxidizing species electrochemically produced to the MB molecule. In fact, this feature is another advantage to use the smartphone protocol in color decay because when the goal is to assess the treatment efficiency of a given technology, the use of UV–vis spectrophotometric methods to quantify, the color removal or/and the concentration of a target organic molecule, should be analyzed carefully, especially when dealing with real matrices and/or advanced oxidation/reduction technologies. It is possible to have spectral interferences by transformation intermediates and matrix components, which may absorb radiation at the wavelength of the target organic pollutant absorption maximum. For this reason, the determinations by using the colored-images obtained by smartphone camera and analyzed, according their surface’s color, allow more accurate results avoiding the absorbace interferences.

It is important to indicate that the electrogenerated oxidants at anodic material, during EO process, promote the degradation of MB and afterwards, it is broken into smaller ones that do not influence the color but remain in the solution, due to their recalcitrance^22–25^. At diamond electrode, this behavior is due to the physisorbed ^•^OH, which are efficiently electrogenerated via water discharge (Eq. 7) at its surface, and confined close to the anode surface in the reaction cage, favoring the degradation of organic matter and, consequently, achieving the best oxidation performances^26^.

7 $${\text{BDD}} + {\text{H}}_{{2}} {\text{O}} \to {\text{BDD}}\left( {^{ \bullet } {\text{OH}}} \right) + {\text{H}}^{ + } + {\text{e}}^{ - }$$

The higher amount of the heterogenous free ^•^OH promotes the complete incineration of pollutants to CO_2_ and water by indirect oxidation approach. However, the electrosynthesis of persulfate and radical sulfate (S_2_O_8_^2−^ and SO_4_^•−^) at diamond films have been described in the literature as one of the most efficient in sulfate medium^27^. S_2_O_8_^2−^ formation, from the EO of sulfate species depends on the properties of the electrode (boron doping, *sp*^2^/*sp*^3^ ratio, roughness, electrode support, thickness), electrolyte medium (sulfuric acid, sulfuric-based acids or sulfate salts) and its concentration (from 0.1 to 1 mol L^−1^) as well as the organic-sulfate salt precursor^27–34^. Two mechanisms have been proposed: direct (Eq. 8) or indirect (Eq. 9) mechanisms are the main routes to electrogenerate S_2_O_8_^2−^^28,30,35–42^. But, considering that SO_4_^•−^ can be generated via reaction with heterogenous free ^•^OH (Eq. 9), which are formed via water discharge (Eq. 7), in the latter.

8 $${\text{2SO}}_{{4}}^{{{2} - }} \to {\text{ S}}_{{2}} {\text{O}}_{{8}}^{{{2} - }} + {\text{ 2e}}^{ - }$$

9 $${\text{SO}}_{{4}}^{{{2} - }} +^{ \bullet } {\text{OH}} \to {\text{2SO}}_{{4}}^{ \bullet - } \to {\text{S}}_{{2}} {\text{O}}_{{8}}^{{{2} - }}$$

In this way a mix of the oxidants can be produced into the reaction cage (diamond electrode-^•^OH/SO_4_^•−^/S_2_O_8_^2−^)^27,28,40,43^; and this system is suitable as *in-situ* or *ex-situ* oxidation approach to eliminate target pollutants or microorganisms in different water matrices^27,28,40,43^ and organics in soil. In fact, the effectiveness of this process is observed when COD decay is determined. As observed in Fig. 6, high COD removals were achieved at all *j* used, but at 90 mA cm^−2^, 80% of removal was reached. MB is a big molecule containing nitrogen, sulfur, and chlorine atoms besides carbon and hydrogen. During the degradation pathway via heterogeneous free •OH and the persulfate and/or sulfate radicals^44^, the molecule is successively broken into smaller ones until it turns probably into NH_4_^+^, SO_4_^2−^, NO_3_^−^, CO_2_ and water^23^.

**Figure 7:**
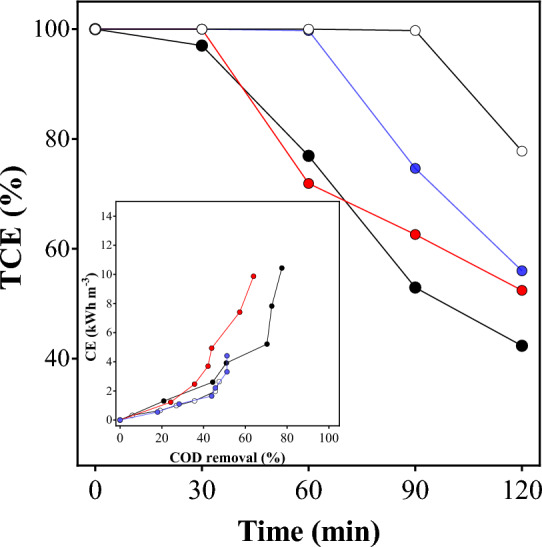
TCE (%) and CE (inset) during electrolysis with 30 (), 45 (), 60 () and 90 mA cm^−2^ ().

Figure 7 shows the TCE evolution (%) for the electrochemical treatment of the MB solution. As can be seen, higher TCE values were obtained when lower *j* were applied, during 60 min of treatment. This behavior is due to the efficient use of electrical charge for the degradation of organic matter in the solution, and after that, it decays linearly in the course of electrolysis. Conversely, %TCE decreased significantly when higher* j* were applied because a significant amount of electrical energy was used in unwanted reactions, decreasing the overall efficiency. Another feature that should be considered is that, the cathodic reactions can influence negatively, as in the case of reduction of the H^+^. Hydrodynamic conditions of the effluent can be also influenced by the flow or agitation conditions at the electrochemical cell. Then, taking into account that, the energy consumption (CE) was also estimated. Inset in the Figure 7 illustrates the CE as a function of the COD removal for each one of the *j* studied.

Although similar COD removals were achieved at all *j*, higher energy requirements were achieved at higher *j* applied. This, as already mentioned, is attributed to a greater contribution from non-oxidative parasitic reactions. However, it is an information that exemplify when the electrochemical treatment could be stopped and coupled with other AOP to enhance the elimination of organics in the solution. The elimination of dye in short times allow to achieve more than 50% of organic matter removals under specific operating conditions, allowing to reduce the effective treatment time.

In the case studied here, both COD and color decay behaviors make sense themselves and together with the abovementioned statements are sufficient to prove the smartphone method's efficacy. However, with a brief literature analysis, it is clear that an equivalent abatement in color and COD is also pointed out by other works, by using the same kind of anode^45^, or even with a different one, as the Ti/RuO_2_–IrO_2_ or SnO_2_^46^. In the recent years, there is a significant effort in increasing the sustainability not only of industrial but also of environmental processes. New concepts arising from both the theory of circular economy and application of life cycle assessment tools have made researchers and technicians to be ready for a change of paradigm in the electrochemically assisted waste remediation technologies^47–50^. Within this framework, several electrochemical innovative approaches are being developed where advanced materials science and digitalization may help achieving sustainable development goals (SDG) in key area focus on clean water and sanitation (SDG 6)^51,52^, and the use of smartphone protocol is a novel initiative that integrated to EAOPs, it can become a real application in the decentralization of analytical procedures through cheaper and portable technologies.

Thus, the efficacy of the proposed-method even in real matrices of water was proved when a sample of raw effluent from a beauty salon was electrochemically treated using BDD as anode, adding or not, 0.04 M of Na_2_SO_4_ as the electrolyte. Then, COD measurements were obtained and compared by the two methods, before and after the treatment. Results are demonstrated in the Table 2.

**Table 2:** Beauty salon COD values (mg L^−1^): comparison between the two methods.

Sample	Spectrophotometer	Smartphone	Average COD	Standard deviation
Raw	1829	1908	1869	40
Raw + 0.1 M Na_2_SO_4_	1205	1274	1240	34
After treatment	903	897	900	3

Despite the standard deviation values obtained, that a first eye can be interpreted as higher deviations (until 40 mg L^−1^), it is important to mention that it only represents around 2% of the total of COD measured, and it should be also considered that the samples were diluted approximately 20 times, then, this deviation value is within the statitistical error described by experts in the field^53^.

## Conclusion

The methods proposed in this work were evaluated under different conditions and achieved good results not only in the calibration curves but also in the electrochemical treatment of water polluted with a high concentration of MB, which proves that the methods can be used even in high-colored matrices of water.

For COD analysis, the average accuracy for the method was 96.2% for the measurements with the spectrophotometer, while that 98.3% for the ones captured by the camera of the smartphone. In the color analysis, it was demonstrated that UV-vis measurement is not feasible to follow the real concentration of the dye in the water and consequently its abatment. Contrary to the huge number of publications, it is clear that the spectrophotometer is not effective when more than 10 mg L^−1^ is used as concentration of dye. With the method here proposed, no dilution has to be done for the concentration of MB until 50 mg L^−1^, once the smartphone is capable to read the difference of RGB values efficiently. The methods were tested with different current densities during EO with BDD as anode, and the results proved to be equivalent to that obtained by spectrophotometer and the others found in the literature^54–56^.

Thus, these methods based on the use of a simple smartphone with a camera can be a promising way for environmental analysis when spectrophotometers are not available, decentralizing the procedures. Better than this, regarding the color analysis, the method proved to be more efficient than the ones based on the analysis of absorbance decay by spectrophotometer since it makes it possible to work with higher concentrations and no necessity of dilution.

Further studies are in progress in order to develop a smartphone app for android system including both analytical determinations, focusing on the digitalization and decentralization of analytical instruments for environmental applications^14^. Also, the use of renewable energies to supply electrical energy in remote cities is being investigated^57,58^.

## Data Availability

We want to formally declare that the datasets generated and/or analysed during the current study are not publicly available due to the elaboration of a patent document, which protect the confidenciatily of the analytical calibration and the production of an algorithm/software to establish a smartphone commercial application, but are available from the corresponding author on reasonable request.
